# Combat PTSD and Implicit Behavioral Tendencies for Positive Affective Stimuli: A Brief Report

**DOI:** 10.3389/fpsyg.2016.00758

**Published:** 2016-05-24

**Authors:** Ashley N. Clausen, Westley Youngren, Jason-Flor V. Sisante, Sandra A. Billinger, Charles Taylor, Robin L. Aupperle

**Affiliations:** ^1^Laureate Institute for Brain Research, TulsaOK, USA; ^2^University of Tulsa, TulsaOK, USA; ^3^University of Kansas Medical Center, Kansas CityKS, USA; ^4^University of California, San DiegoCA, USA; ^5^University of Missouri-Kansas City, Kansas CityMO, USA

**Keywords:** combat PTSD, veterans, inhibition, approach behavior, avoidance behavior

## Abstract

**Background:** Prior cognitive research in posttraumatic stress disorder (PTSD) has focused on automatic responses to negative affective stimuli, including attentional facilitation or disengagement and avoidance action tendencies. More recent research suggests PTSD may also relate to differences in reward processing, which has lead to theories of PTSD relating to approach-avoidance imbalances. The current pilot study assessed how combat-PTSD symptoms relate to automatic behavioral tendencies to both positive and negative affective stimuli.

**Method:** Twenty male combat veterans completed the approach-avoidance task (AAT), Clinician Administered PTSD Scale, Beck Depression Inventory-II, and State-Trait Anger Expression Inventory-II. During the AAT, subjects pulled (approach) or pushed (avoid) a joystick in response to neutral, happy, disgust, and angry faces based on border color. Bias scores were calculated for each emotion type (avoid-approach response latency differences). Main and interaction effects for psychological symptom severity and emotion type on bias score were assessed using linear mixed models.

**Results:** There was a significant interaction between PTSD symptoms and emotion type, driven primarily by worse symptoms relating to a greater bias to avoid happy faces. *Post hoc* tests revealed that veterans with worse PTSD symptoms were slower to approach as well as quicker to avoid happy faces. Neither depressive nor anger symptoms related to avoid or approach tendencies of emotional stimuli.

**Conclusion:** Posttraumatic stress disorder severity was associated with a bias for avoiding positive affective stimuli. These results provide further evidence that PTSD may relate to aberrant processing of positively valenced, or rewarding stimuli. Implicit responses to rewarding stimuli could be an important factor in PTSD pathology and treatment. Specifically, these findings have implications for recent endeavors in using computer-based interventions to influence automatic approach-avoidance tendencies.

## Introduction

Posttraumatic stress disorder (PTSD) is characterized by intrusion, hypervigilance, negative alterations in mood and cognitions, and avoidance in response to distressing and/or trauma-related stimuli and situations (American Psychiatric Association, 2013). Anhedonia is another facet associated with PTSD, subsumed under negative alterations in mood and cognitions (American Psychiatric Association, 2013), and is often associated with decreased responsivity to reward or positive affective stimuli (Nawijn et al., 2015). PTSD has also been associated with increased anger (McHugh et al., 2012), which has been related to *greater* striatal activation to reward (Pietrini et al., 2000). It is suggested that PTSD is associated with deficits in the approach and avoidance systems that underlie reward responsivity, as well as avoidance of trauma-related stimuli (Stein and Paulus, 2009). For example, emotional numbing, anhedonia, and impulsivity (James et al., 2014) have been associated with dysregulation of approach systems, whereas avoidance, hyperarousal and irritability have been associated with dysregulation of the avoidance system (Stein and Paulus, 2009). Neurocognitive research aimed at understanding potential approach-avoidance imbalances may be important for informing the treatment of PTSD. Specifically, understanding the extent to which approach-avoidance imbalances may be driven by automatic, or implicit, tendencies (versus processes under more explicit cognitive control) has implications concerning the types of interventions which may be effective at restoring this balance.

Neurocognitive research related to PTSD has focused primarily on responses to negative or trauma-relevant affective stimuli. Results from research investigating implicit attentional processes suggest that individuals with PTSD may experience greater attentional interference by negative or trauma-relevant affective stimuli (Fani et al., 2012). In other words, trauma-related or negative stimuli disrupt their attentional focus on aspects of the environment that are more relevant to the goal at hand. Fani et al. (2012) suggest that this attentional interference may be an adaptive behavior in threatening situations by increasing situational awareness. However, attentional interference in non-threatening situations (e.g., social situations) may also be maladaptive, may interfere with an individual’s quality of life, and may lead to a decreased ability to process other situation-relevant information (e.g., safety signals), which may consequently lead to the exaggerated fear response associated with PTSD (Fani et al., 2012).

The approach-avoidance task (AAT) was developed to identify automatic *behavioral* tendencies to approach or avoid affective stimuli (Rinck and Becker, 2007). Subjects respond to stimuli by pulling a joystick toward (approach) or pushing a joystick away from themselves (avoidance; Rinck and Becker, 2007) based on a colored border. Differences in reaction times to “approach” or “avoid” stimuli of different valences can provide an estimate of response tendencies. This task has been used to identify tendencies to avoid affective stimuli in social anxiety (utilizing emotional facial expressions; Heuer et al., 2007; Lange et al., 2008), and spider phobia (utilizing spider images; Klein et al., 2011). Results from these studies suggest a bias to avoid threatening stimuli (Heuer et al., 2007; Rinck and Becker, 2007; Lange et al., 2008; Klein et al., 2011) and positive emotional faces (Heuer et al., 2007).

There have been two studies utilizing the AAT to investigate behavioral tendencies for individuals with PTSD associated with trauma-related, negative stimuli (Fleurkens et al., 2014; Wittekind et al., 2015). One study used the AAT to assess behavioral tendencies for individuals with PTSD related to sexual assault, and reported a tendency to avoid trauma-relevant stimuli (threatening sexual situation images; Fleurkens et al., 2014). Fleurkens et al. (2014) also reported depressive symptom severity specifically to relate to increased avoidance tendencies for positively valenced non-threatening and non-trauma related images (e.g., flowers). The most recent study examining behavioral tendencies in PTSD included adults who were displaced as children following the end of World War II. This study reported no differences in behavioral tendencies to trauma-related (e.g., war-related) stimuli between those with and without a PTSD diagnosis (Wittekind et al., 2015). Thus, there is inconsistent support in the literature that PTSD relates to automatic avoidance tendencies in response to trauma-relevant images and initial evidence that depressive symptoms may relate to avoidance tendencies for positive affective stimuli. While inconsistencies across studies may be due to differences in trauma type, symptom severity, or stimuli utilized in the task, the small number of studies conducted in this area precludes determination of how these factors may impact results.

Investigations using trauma-unrelated affective stimuli (e.g., emotional faces) are important for understanding whether observed behavioral tendencies may generalize to other situations and contexts. The investigation of behavioral tendencies to positively valenced stimuli or situations would also be important, as this could relate not only to underlying symptoms of anhedonia or emotional numbing, but perhaps also to motivation for treatment. There have been recent endeavors to use computer-based interventions (Amir et al., 2009) to modify automatic tendencies and benefit symptoms for various mental health populations (Mathews and MacLeod, 2002; Wilson et al., 2006; Hakamata et al., 2010). Additionally, research has begun to investigate the use of a modified AAT as an intervention to specifically target approach tendencies toward positive stimuli, and benefit symptoms of social anxiety disorder (Taylor and Amir, 2012; Asnaani et al., 2014). Results from this line of research suggest that time-limited training that targets approach behavior may increase approach tendencies (Asnaani et al., 2014), and engagement in social situations (Taylor and Amir, 2012). However, little is known about the potential impact of such interventions for treatment of PTSD. Further understanding of approach-avoidance behavioral tendencies in PTSD would have implications for the viability of such interventions for targeting approach tendencies or interpersonal deficits associated with PTSD (King et al., 2006).

In the current study, we examined approach and avoidance tendencies in response to trauma-unrelated affective stimuli (emotional faces). This choice of stimuli was to identify whether PTSD relates to differences in approach and avoidance tendencies that are pervasive in generalizing to trauma-unrelated stimuli and situations. In addition, we aimed to understand how PTSD may relate to approach and avoidance tendencies to both positive and negative affective stimuli, which would be difficult to do if focusing exclusively on trauma-related stimuli. The use of emotional faces could also have implications for understanding interpersonal deficits that are commonly associated with PTSD (King et al., 2006). We hypothesized that PTSD symptoms would relate to increased avoidance tendencies to negative and positive affective stimuli, similar to previous research in PTSD and anxiety disorder populations (Rinck and Becker, 2007; Fleurkens et al., 2014; Wittekind et al., 2015). We also hypothesized that depressive symptoms would relate to increased avoidance tendencies to positive affective stimuli (Trew, 2011), given consistent findings in previous research that depressive symptoms relate to deficits in processing of reward and positive affective stimuli (Admon and Pizzagalli, 2015). Lastly, we hypothesized that anger symptoms would relate to increased approach tendencies toward all affective stimuli, given theories that anger is an approach-motivated emotion (Carver and Harmon-Jones, 2009).

## Materials and Methods

### Subjects

Subjects included 20 male veterans (mean age = 32.75, *SD* = 7.69; mean education = 15.55, *SD* = 1.85) with varying PTSD symptomology (10 met diagnostic criteria for PTSD, and 10 did not meet diagnostic criteria for PTSD, as determined via the Clinician Administered PTSD Scale; Blake et al., 1995) who served in combat since the onset of Operation Iraqi Freedom in March 2003. Participants were recruited as part of a larger study examining cardiovascular health in veteran populations. Therefore, inclusion and exclusion criteria were chosen to not only limit confounding variables for the current investigation but also to limit potential confounds of cardiovascular response for the larger study. Participants were excluded from the current study if they reported any of the following: non-veteran status, psychiatric symptoms requiring immediate mental health attention, substance or alcohol abuse or dependence over the past 6 months, diagnosis of any medical condition that directly affects cardiovascular function (i.e., stroke, peripheral, or atherosclerotic vascular disease), history of moderate to severe head injury (loss of consciousness >30 min and/or post- traumatic amnesia > 1 day), or history of neurological disorder, or use of any medications within the last 30 days that may impact brain function (i.e., hypertensive medications; stimulants). Participants taking a stable dose of psychotropic medication for at least two consecutive months were included in the present study; however, none of the enrolled participants reported currently taking psychotropic medications. Participants were also excluded if they reported being diagnosed, or met diagnostic criteria for, as determined via the Mini International Neuropsychiatric Inventory (Sheehan et al., 1998), schizophrenia or bipolar I disorder. Additionally, participants who reported a psychological disorder other than PTSD as the primary cause for distress were excluded.

This study was approved by the University of Kansas Medical Center and the University of Missouri – Kansas City Institutional Review Boards. Written, signed consent was obtained from all subjects prior to completion of study procedures.

### Psychological Assessment

Recruitment and study procedures began prior to dissemination of the DSM-5. Therefore, all psychodiagnostic assessment was based on DSM-IV criteria. PTSD symptoms were assessed using the [Clinician Administered PTSD Scale (CAPS) – IV; Blake et al., 1995] and were based on combat-related Criterion A events. The CAPS is a semi-structured diagnostic interview, which was administered by a clinical psychology doctoral student (ANC), and supervised by a licensed clinical psychologist (RLA). The past month total score (intensity + frequency) was used as the primary outcome. The Anger Expression Index of the State Trait Anger Expression Inventory-II (STAXI-II) was used to assess state and trait total anger expression, and/or suppression (Spielberger, 1999). The Beck Depression Inventory (BDI)-II (Beck et al., 1996) total score was used to assess depressive symptoms over the previous 2 weeks.

### Approach-Avoidance Task

The AAT was conducted similarly to previously described tasks (Najmi et al., 2010; Taylor and Amir, 2012), and included images of eight actors (four female, four male) displaying happy, neutral, angry, and disgust faces from the NimStim Set of Facial Expressions (Tottenham et al., 2009). Subjects were instructed to move a joystick toward or away from them based on the colored borders surrounding each image. Subjects pulled the joystick toward them if the border was green and pushed the joystick away from them if the border was blue, responding only to the border color. Subjects completed 12 practice and 128 assessment trials (eight trials for each facial expression in the approach condition, and eight trials for each facial expression in the avoidance condition) with different randomized orders of actor, expression, and border color for each subject. Images became increasingly larger or smaller as the subjects pulled or pushed the joystick, respectively, until the image disappeared (when the joystick reached a 30° position). Response latencies were calculated based on the time the image remained on the screen. Incorrect trials (1.9%) were excluded from the analyses. Bias scores for each facial expression (happy, neutral, angry, and disgust) were calculated using median response latencies to push versus pull trials and denoted, for example, avoid_a_-approach_a_ for angry conditions. More negative values reflect greater avoidance bias whereas larger positive scores reflect greater approach tendencies.

### Statistical Analyses

Statistical analyses were conducted using R Statistical Package^1^. Age and education were explored as possible covariates using Spearman’s rho. Three linear mixed effects analyses (LMEs) were used to investigate main and interaction effects for each psychological measure (PTSD, depression, and anger) and emotion type (happy, neutral, angry, and disgust) on avoid-approach bias scores. While effect sizes for LME’s are controversial in the present literature given the impact and difficulty estimating random effects, conditional effects sizes for LME models were estimated. Results were considered significant at *p* < 0.05, Bonferroni corrected for three LMEs (adjusted *p*-value threshold of *p* < 0.017).

*Post hoc* Spearman’s rho correlations were used to clarify LME results and identify potential relationships between the avoid-approach bias scores for each specific emotion type and psychological symptom severity including PTSD, depressive, and anger symptoms. To further delineate identified effects of implicit approach and avoidance tendencies, a second bias score was calculated (Najmi et al., 2010; Taylor and Amir, 2012). The second bias score was used to examine the approach (pull) and avoidance (push) in emotionally valenced conditions separately, relative to the neutral condition (i.e., push_a_–push_n_).

## Results

Subjects endorsed mild levels of PTSD (CAPS = 30.10, *SD* = 19.01), depression (BDI-II = 9.1, *SD* = 7.8) and anger symptoms (STAXI-II = 40.11, *SD* = 12.20). Neither age nor education correlated with avoid-approach bias scores (all *p*’s > 0.07).

**FIGURE 1:**
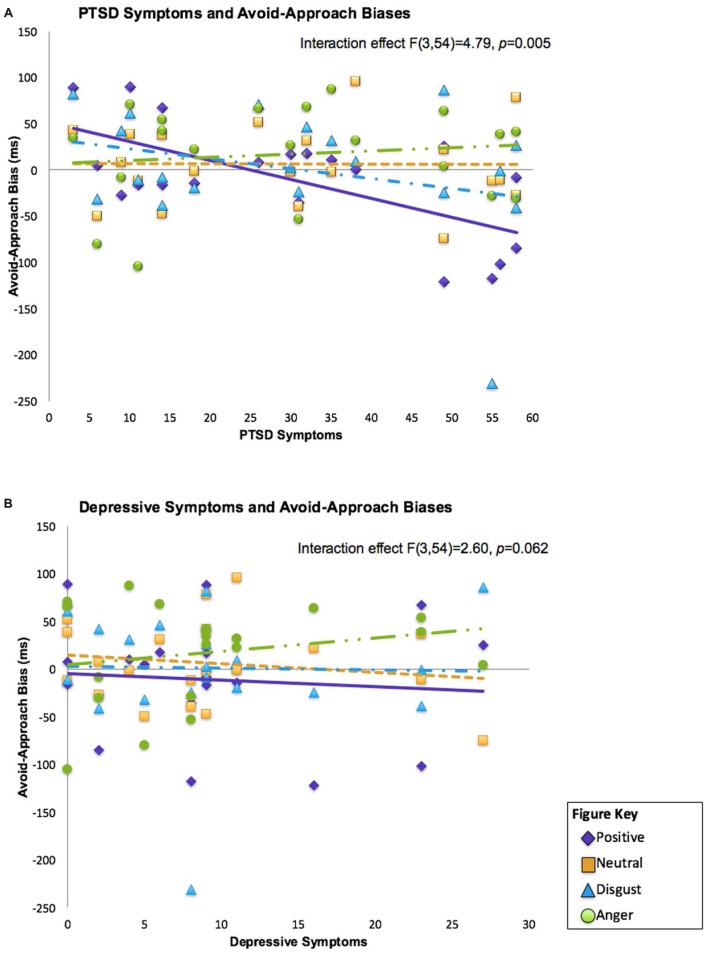
**Relationships between psychological symptoms of posttraumatic stress disorder (PTSD; **A)** and depression **(B)**, and avoid-approach biases of emotionally valenced faces (positive, neutral, angry, and disgust) in combat veterans.** PTSD symptoms were significantly related to avoid-approach biases of positive stimuli. Results from the linear mixed model interaction effect are presented.

Linear mixed effects analyses analyses revealed a significant interaction with a medium effect between PTSD symptoms and emotion type [*F*(3,54) = 4.80, *p* = 0.005, conditional effect Cohen’s *d* = 0.518], but no main effect for either PTSD symptoms [*F*(1,18) = 2.05, *p* = 0.692] or emotion type [*F*(3,54) = 1.62, *p* = 0.195]. The interaction between depressive symptoms and emotion type was trending with a small to medium effect [*F*(3,54) = 2.60, *p* = 0.0617, conditional effect Cohen’s *d* = 0.471], but there was no main effect for depressive symptoms or emotion type [*F*(1,18) = 0.19, *p* = 0.692; *F*(3,54) = 1.46, *p* = 0.235; respectively]. There were no main or interaction effects with anger symptoms, yielding small effects (all *p*s > 0.10, conditional effect Cohen’s *d* = 0.407). **Figure 1** depicts relationships between PTSD and depressive symptoms and approach/avoidance tendencies by emotion type.

*Post hoc* correlations indicated that individuals with worse PTSD symptoms exhibited a greater avoid_h_-approach_h_ bias (greater avoidance) for happy faces, and demonstrated a large effect (ρ = -0.45, *p* = 0.046; all other *p*’s > 0.10). Despite a trending interaction between depressive symptoms and emotion type, there were no significant relationships between depressive symptoms and avoid-approach bias scores (all *p*’s > 0.10).

To further characterize the relationship between PTSD symptoms and the avoid_h_-approach_h_ bias score, we examined the individual bias scores for happy faces for approach and avoidance response directions separately compared to neutral faces (i.e., push_h_–push_n_; pull_h_–pull_n_). Large effects were found for both approach and avoidance conditions, suggesting that those with worse PTSD symptoms were slower to approach (pull_h_–pull_n_; ρ = -0.492, *p* = 0.028), and quicker to avoid (push_h_–push_n_; ρ = 0.543, *p* = 0.013) happy faces.

## Discussion

In the present pilot study, combat-PTSD symptoms related to both decreased approach and increased avoidance tendencies in response to positive affective stimuli (happy faces). These findings are consistent with reports of decreased behavioral and neural (striatal) responsivity to reward in PTSD (Nawijn et al., 2015). Our results extend previous work by providing evidence that PTSD relates to both decreased approach and increased avoidance tendencies in response to positively valenced stimuli, and that this effect may be better accounted for by PTSD versus depressive symptoms. While prior research using the AAT in anxiety and depression has identified *avoidance* tendencies to positive affective stimuli (Heuer et al., 2007; Fleurkens et al., 2014), the present results suggest both avoidance and approach tendencies related to positive affective stimuli may be important for the conceptualization and treatment of PTSD symptoms.

In previous research, PTSD diagnosis has been reported to relate to greater attentional biases for negative affective stimuli (Fani et al., 2012). The few studies that have used the AAT to examine automatic approach-avoidance tendencies related to PTSD have been mixed, with one reporting greater avoidance tendencies to trauma-related stimuli and another reporting no differences (Fleurkens et al., 2014; Wittekind et al., 2015). In the present study, PTSD symptoms were not related to behavioral tendencies for negative affective stimuli. This may have been due to the negative affective stimuli (angry and disgust faces) not being as salient as trauma-related stimuli or to the limited sample size of the current study. This would suggest that approach-avoidance tendencies to negative affective stimuli may not be pervasive in extending to trauma-irrelevant stimuli. It is also possible that approach-avoidance tendencies are state, rather than trait, measures that depend upon the current context and emotional state of the individual. This would make for a relatively unreliable measure that causes different studies to identify conflicting results. In support of this hypothesis, one study assessing the psychometric properties of the AAT suggests relatively low test–retest reliability (*r* = 0.35; Reinecke et al., 2010). Therefore, it would be important for future research to assess approach-avoidance behavior with state and trait measures to provide a clearer picture of implicit tendencies associated with PTSD symptomology.

In the present study, individuals with greater PTSD symptom severity exhibited behavioral biases associated with positive affective stimuli (happy faces). Our results suggest that avoidance tendencies to *positive* affective stimuli may be more robust than to negative affective social stimuli in PTSD, and warrant further investigation. In particular, further research is needed to understand whether the avoidance tendencies to positive affective stimuli are specific to the social domain (facial stimuli) or if it generalizes to other domains. Notably, one previous study reported depressive symptom severity reported by individuals with PTSD to relate to increased avoidance of more general positive affective stimuli (i.e., images of flowers). While our findings related specifically to PTSD symptoms and not depressive symptom severity, both of these findings suggest that implicit behavioral tendencies to positive affective stimuli play a role in trauma-related psychological symptoms.

Evidence-based treatments for PTSD (i.e., Prolonged Exposure therapy; Foa, 1997; Foa et al., 2005) typically target avoidance behavior in response to distressing or anxiety-provoking situations. Results from the present pilot study suggest that individuals with increased PTSD symptoms may also have a greater tendency to avoid positive interactions or situations, which are not specifically targeted in evidence-based PTSD treatments. However, further research is needed to determine whether currently supported treatments for PTSD that emphasize trauma-related stimuli or situations (i.e., Prolonged Exposure, Cognitive Processing Therapy) influence automatic behavioral tendencies to positive affective stimuli or whether trauma-unrelated interventions that specifically target engagement with rewarding or reinforcing activities and situations (i.e., Behavioral Activation; Martell et al., 2013), positive affect (Sin and Lyubomirsky, 2009) or positive social interactions, would provide additional benefit to evidence-based trauma treatment. While some evidence-based PTSD treatments incorporate aspects of pleasant activity scheduling, stand-alone treatments such as Behavioral Activation more explicitly target engagement in naturally reinforcing or fulfilling behaviors. In addition, future research is needed to identify whether such tendencies can be modified through implicit training, for example by using a modified AAT in which the majority of the trials require “approaching” positive affective stimuli (Jakupcak et al., 2006; Taylor and Amir, 2012).

The current study was limited by a small sample and the lack of a non-trauma exposed group. Furthermore, all subjects were male combat veterans. Therefore, generalizability to other populations is unknown. In addition, subjects in the present study endorsed mild to moderate levels of PTSD symptomology. This limits generalizability to more severe PTSD populations and may provide a reasonable explanation for the lack of expected results related to negatively valenced stimuli. However, this also suggests that automatic approach-avoidance imbalances may be evident even for those with only moderate symptom levels. Nevertheless, utilizing a sample with more severe PTSD symptom severity or the use of trauma-relevant stimuli might yield different results and provide additional insights into the role of approach and avoidance behavior in PTSD populations. It is important for future, larger studies to investigate the impact of a broader range of PTSD symptomology and to examine the contribution of individual PTSD symptom clusters on approach and avoidance behavioral tendencies. Despite limitations, the current results provides initial evidence that PTSD symptoms are associated with differences in implicit behavioral tendencies in response to positively valenced stimuli, characterized by both reduced approach and increased avoidance tendencies. These results highlight the potential value of targeting responses to positive affective stimuli in PTSD treatment.

## Author Contributions

AC: Substantial contributions to the conception and design of the work; data acquisition, data analysis and interpretation of results. Contributions to drafting, and revising the work critically for important intellectual content. Provided final approval of the version to be published, and agreement to be accountable for all aspects of the work in ensuring that questions related to the accuracy or integrity of any part of the work are appropriately investigated and resolved. WY: Substantial contributions to data analysis. Revising the work critically for important intellectual content. Provided final approval of the version to be published, and agreement to be accountable for all aspects of the work in ensuring that questions related to the accuracy or integrity of any part of the work are appropriately investigated and resolved. J-FS: Substantial contribution to data acquisition. Revising the work critically for important intellectual content. Provided final approval of the version to be published, and agreement to be accountable for all aspects of the work in ensuring that questions related to the accuracy or integrity of any part of the work are appropriately investigated and resolved. SB: Substantial contributions to the conception and design of the work. Revising the work critically for important intellectual content. Provided final approval of the version to be published, and agreement to be accountable for all aspects of the work in ensuring that questions related to the accuracy or integrity of any part of the work are appropriately investigated and resolved. CT: Substantial contributions to data analysis and interpretation of results. Revising the work critically for important intellectual content. Provided final approval of the version to be published, and agreement to be accountable for all aspects of the work in ensuring that questions related to the accuracy or integrity of any part of the work are appropriately investigated and resolved. RA: Substantial contribution to the concept or design of the work, and interpretation of results. Revising the work critically for important intellectual content. Provided final approval of the version to be published, and agreement to be accountable for all aspects of the work in ensuring that questions related to the accuracy or integrity of any part of the work are appropriately investigated and resolved.

## Conflict of Interest Statement

The authors declare that the research was conducted in the absence of any commercial or financial relationships that could be construed as a potential conflict of interest.

## Footnotes

1 https://cran.r-project.org
